# TEMPO-Oxidized Cellulose Hydrogels Loaded with Copper Nanoparticles as Highly Efficient and Reusable Catalysts for Organic Pollutant Reduction

**DOI:** 10.3390/gels11070512

**Published:** 2025-07-01

**Authors:** Yangyang Zhang, Yuanyuan Li, Xuejun Yu

**Affiliations:** 1 Bamboo Industry Institute, Zhejiang A&F University, Hangzhou 311300, China; zhangyangyang@zafu.edu.cn (Y.Z.); yuanli@zafu.edu.cn (Y.L.); 2 National Key Laboratory for Development and Utilization of Forest Food Resources, Zhejiang A&F University, Hangzhou 311300, China

**Keywords:** cellulose hydrogels, Cu nanoparticles, TEMPO-mediated oxidation, reusable catalyst

## Abstract

To successfully prepare cellulose hydrogels through a dissolution–regeneration process, 60 wt% LiBr aqueous solution was used as a green solvent. Carboxyl groups were precisely introduced onto the surface of the cellulose hydrogels through a TEMPO-mediated oxidation reaction, while the three-dimensional network structure and open pore morphology were completely retained. This modification strategy significantly enhanced the loading capacity of the hydrogels with copper nanoparticles (Cu NPs). The experimental results show that the LiBr aqueous solution can efficiently dissolve cellulose, and the TEMPO oxidation introduces carboxyl groups without destroying the stability of the hydrogels. Cu NPs are uniformly dispersed and highly loaded on the surface of the hydrogel because of the anchoring effect of the carboxyl groups. Cu NP-loaded hydrogels exhibit excellent catalytic activity in the NaBH_4_ reduction of 4-nitrophenol (4-NP). Cu NP-loaded hydrogels maintain their complete structure and good catalytic performance after five consecutive cycles. Moreover, Cu NP-loaded hydrogels demonstrate high efficiency in degrading organic dyes such as methyl orange and Congo red. This study successfully developed efficient, low-cost, and environmentally friendly Cu NP-loaded hydrogel catalysts through the synergistic effect of LiBr green solvent and TEMPO oxidation modification, providing a feasible alternative to noble metal catalysts.

## 1. Introduction

Noble metal nanostructures (such as Pd, Pt, Au, and Ag) have attracted considerable attention in various fields, including catalysts [1,2,3], sensors [4,5], and biomedicine [6,7]. Notably, noble metal nanoparticles exhibit excellent catalytic activity in water purification processes via catalytic degradation and have been widely employed in both industrial and academic settings [8,9,10,11,12,13,14,15], for example, Pd, Pt, Au, and Ag. However, the high cost (Pd: ~$30–110/g; Pt: ~$25–42.5/g; Au: ~$50–120/g; and Ag: $0.5–1.2/g, data from the Daily Metal Price for the last 5 years, 2020–2025) and scarcity of most noble metals severely limit their widespread application [16,17]. In comparison, copper (Cu), due to its abundant natural occurrence and low cost ($0.006–0.012/g, data from the Daily Metal Price for the last 5 years, 2020–2025), has been regarded as a promising alternative [18,19,20]. Nevertheless, a major limitation of using metal nanoparticles in catalytic degradation for water purification is their tendency to aggregate, which arises from their high surface energy. This aggregation often results in reduced catalytic activity and stability [9,14,19]. Moreover, these nanoparticles typically exist in powder form, making them difficult to recycle and thus leading to low reusability [13,14]. These challenges can be effectively addressed by immobilizing metal nanoparticles onto a supporting substrate.

An attractive class of supporting substrates is hydrogels, which possess highly porous network structures and large specific surface areas, enabling the in situ synthesis of nanoparticles [21,22,23]. Sultan et al. reported the synthesis of a hydrogel based on acrylamidoglycolic acid (AAGA) and its use as a supporting substrate in the preparation of Cu nanoparticles [24]. Godiya et al. developed a bio-based bilateral hydrogel from carboxymethyl cellulose (CMC) and polyacrylamide (PAM), which was loaded with in situ-reduced Cu nanoparticles for catalytic applications [25]. These Cu nanoparticle-loaded hydrogels exhibited high catalytic efficiency for nitrophenols and other organic dyes. However, the toxic crosslinking agents and petrochemical-derived monomers used in their hydrogel preparation may pose environmental and health risks. Cellulose, the most abundant natural polymer on Earth, offers numerous advantages, such as renewability, sustainability, biocompatibility, and biodegradability. It has been widely explored in the field of hydrogel production. Among them, the dissolution–regeneration method is one of the most common preparation methods [26,27]. In the preparation of cellulose hydrogels, choosing an appropriate solvent system is a key step to achieve cellulose dissolution and subsequent gelation. Commonly used cellulose solvents include N-methylmorpholine-N-oxide (NMMO), ionic liquids (ILs), and LiCl/DMAc systems. These systems offer significant potential for the fabrication of cellulose hydrogels. However, these solvents exhibit certain limitations, such as toxicity, challenges in recycling, high costs, and process instability. While alkali/urea systems can overcome these limitations, they require precooling to −12 °C to achieve dissolution. Recently, a green, low-cost, and recyclable cellulose solvent, lithium bromide (LiBr) solution, has been demonstrated to effectively dissolve cellulose for the preparation of cellulose-based hydrogels. They do not require a coagulation solvent; the cellulose solutions are cooled until solidification when preparing the hydrogel [28,29,30]. The preparation of cellulose-based hydrogels using a LiBr aqueous solution retains a highly nano-porous structure and large surface area. These characteristics make this type of cellulose hydrogel an appealing candidate for nanoparticle anchoring.

Based on the above considerations, herein, cellulose hydrogels were prepared through a dissolution–regeneration process using LiBr aqueous solution. TEMPO-mediated oxidation reaction was employed to introduce carboxyl groups onto the surface of the cellulose hydrogel. We fabricated a series of Cu NP-loaded cellulose hydrogels with varying TEMPO-oxidation times. The catalytic performance and reusability of the Cu NP-loaded cellulose hydrogels were evaluated for the reduction of 4-nitrophenol (4-NP) to 4-aminophenol (4-AP) using NaBH_4_. We also chose organic dyes such as methylene orange and Congo red as another test to examine the universality of Cu NP-loaded cellulose hydrogels.

## 2. Results and Discussion

### 2.1. Basic Properties of Tempo-Oxidized Cellulose Hydrogel

The FTIR spectra of the regenerated cellulose hydrogels closely resembled those of raw cellulose as follows (Figure S1): 3700–3000 cm^−1^ (O–H stretching vibration), 1370 cm^−1^ (C–H asymmetric deformation), 1158 cm^−1^ (C–O–C symmetric bending), and 898 cm^−1^ (β-glucoside bonds between sugar units). Two small bands at 3488 and 3441 cm^−1^ in the O–H stretching region represented the features indicative of regenerated cellulose, cellulose II crystal structure [31,32]. This indicates that no significant chemical changes occurred during dissolution and regeneration.

**Figure 1:**
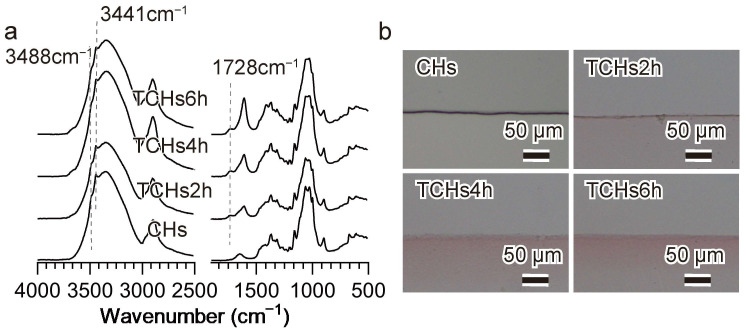
(**a**) FTIR spectra of cellulose hydrogels (CHs) and cellulose hydrogels oxidized for 2, 4, and 6 h (TCHs2h, TCHs4h, and TCHs6h, respectively). (**b**) Light-microscope images of cellulose hydrogels, the colored region corresponds to the localization of ruthenium red, which selectively stains the carboxyl group.

The FTIR spectra of the initial cellulose hydrogels (CHs) and the oxidized cellulose hydrogels (TCHs2h, TCHs4h, and TCHs6h) are shown in Figure 1a. Two small bands in the O–H stretching region represented the features indicative of regenerated cellulose, cellulose II crystal structure. This indicates that the oxidation process did not change the crystalline structure of cellulose. The C=O stretching vibration at 1728 cm^−1^ was observed in the spectra of all oxidized cellulose hydrogels, indicating the introduction of the carboxyl group [33]. The absorbance of this band increased with increasing oxidation time. The carboxyl group content in the oxidized cellulose hydrogels were measured by the conductometric titration method, 0.22 mmol/g for TCHs2h, 0.39 mmol/g for TCHs4h, and 0.50 mmol/g for TCHs6h.

Figure 1b shows the microstructure of cellulose hydrogels stained with ruthenium red. The oxidized cellulose hydrogels (TCHs2h, TCHs4h, and TCHs6h) exhibited a red-stained section, and red color intensity increased with the carboxyl group content. This is because ruthenium red selectively stains carboxyl groups. Notably, the red-stained section was only found in the periphery of the gels, demonstrating that oxidation occurred only on the surface of the cellulose hydrogels.

### 2.2. Synthesis and Characterization of Cu NP-Loaded Cellulose Hydrogel

**Figure 2:**
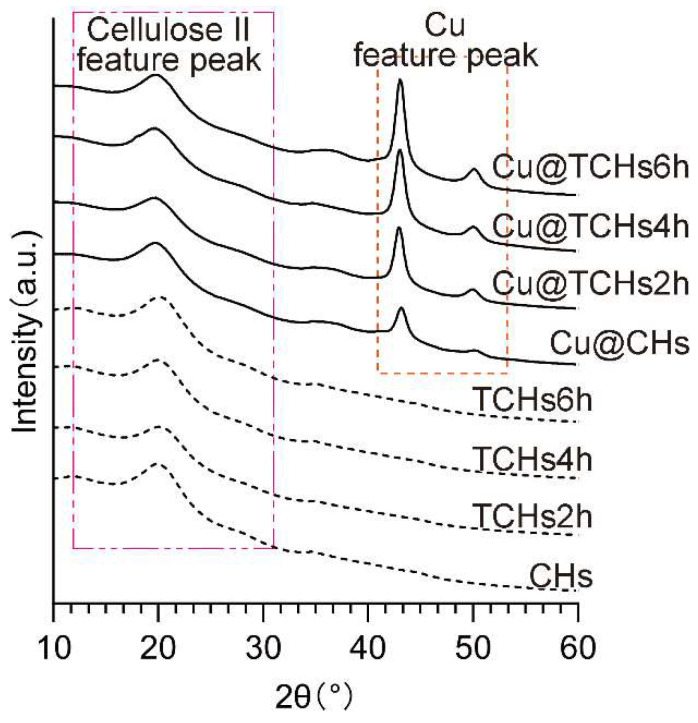
XRD profiles of unoxidized and oxidized cellulose hydrogels and those loaded with Cu NPs.

The diffraction profiles after oxidation were very similar to that of the unoxidized cellulose hydrogels (CHs), presenting a broad halo pattern with an intensity maximum at 2θ = 20.2° (Figure 2). This indicates that the cellulose hydrogels are those typical of cellulose II, even if carboxyl groups were introduced on the surface [29]. This result is consistent with the FTIR results.

The diffraction profiles of the Cu NP-loaded cellulose hydrogels (Cu@CHs Cu@TCHs2h, Cu@TCHs4h, and Cu@TCHs6h) were almost identical to those of the unoxidized and oxidized cellulose hydrogels. However, there are two small but distinct peaks at 2θ = 43.4 and 55.0°, corresponding to the 1 1 1 and 2 0 0 diffractions of Cu, respectively [34], and the intensity of the distinct peaks of Cu increased as the oxidation time and carboxyl content increased. This is because the increased carboxyl group content provides additional Cu^2+^ coordination/adsorption sites, promoting the generation of more nuclei during the NaBH_4_ reduction process and ultimately facilitating Cu nanoparticle formation.

**Figure 3:**
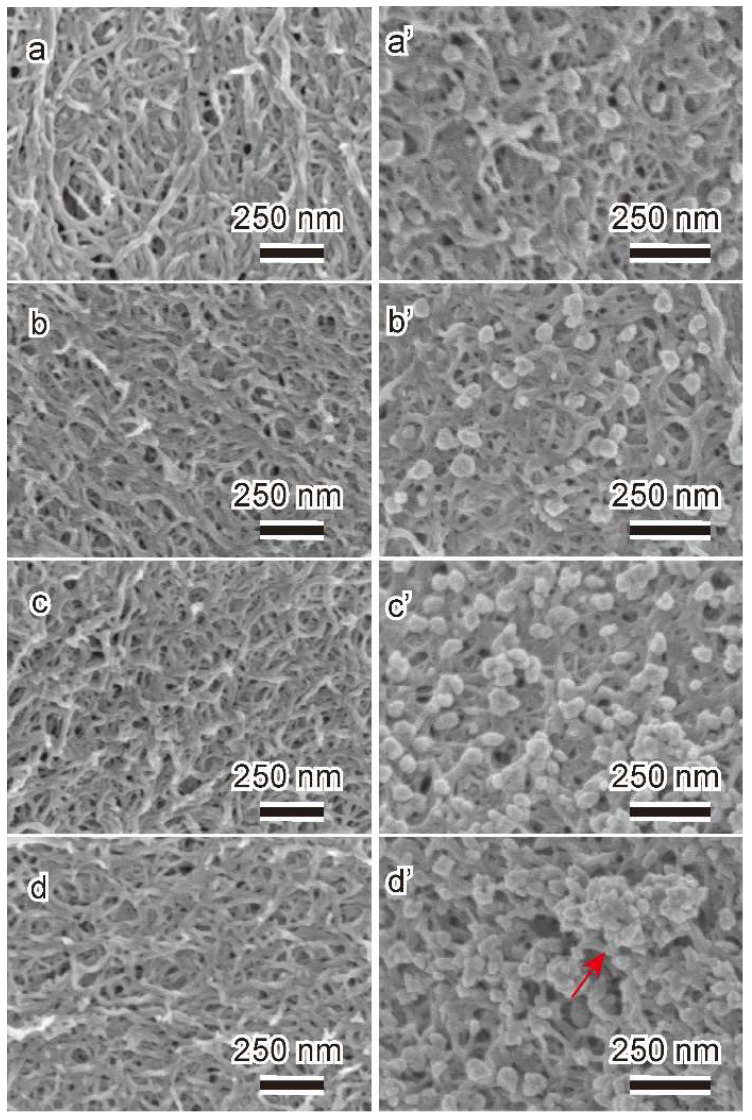
SEM images of the surface section of cellulose hydrogels before and after loading with Cu, (**a**): CHs; (**b**): TCHs2h; (**c**): TCHs4h; (**d**): TCHs6h; (**a’**): Cu@CHs; (**b’**): Cu@TCHs2h; (**c’**): Cu@TCHs4h; and (**d’**): Cu@TCHs6h.

Figure 3 shows the SEM images of the surface section of cellulose hydrogels before and after loading with Cu NPs. The surface of cellulose hydrogel (CHs, TCHs2h, TCHs4h, and TCHs6h) before loading with Cu NPs exhibits a three-dimensional network of nanofibers (width: 20–30 nm) (Figure 3a–d). By introducing a carboxyl group, the fiber structure on the surface of the gel becomes coarse (Figure 3a–d), while there is not much difference in internal fiber structure (Figure S2). This indicates that in the surface region, the partial dissolution of cellulose occurred because of the water solubility of cellouronic acid (carbonylated cellulose at the C6 position). The sectional surface of Cu NP-loaded cellulose hydrogel (Cu@CHs, Cu@TCHs2h, Cu@TCHs4h, and Cu@TCHs6h) exhibits dense granular protrusions with a narrow particle size distribution (10 nm to 120 nm) (Figure 3a’–d’), predominantly displaying near-spherical morphology. This indicates that Cu^2+^ were successfully reduced to form Cu nanoparticles through adsorption, followed by reduction reactions. Notably, the number of Cu nanoparticles increased with an increase in oxidation time and carboxyl group content. However, elevated carboxyl group content and prolonged oxidation time also lead to intensified nanoparticle agglomeration, as indicated by the red arrows in Figure 3d’. To further confirm the incorporation of Cu into cellulose hydrogel, EDS element mapping analysis for the surface of Cu@TCHs4h was carried out, as shown in Figure S3. The results clearly indicate that Cu, C, and O elements are uniformly distributed along the cellulose hydrogel, suggesting the existence of Cu into the surface of Cu@TCHs4h. The amount of Cu nanoparticles loaded the cellulose hydrogels was determined by thermogravimetric analysis (TGA) conducted in N_2_ (Figure S4) With the addition of carboxyl group content to the cellulose hydrogel by oxidation, the amount of Cu nanoparticles in the cellulose hydrogels (dried cellulose gels) is 9.5% for Cu@CHs, 16.3% for Cu@TCHs2h, 19.7% for Cu@TCHs4h, and 25.5% for Cu@TCHs6h.

### 2.3. Catalytic Performance of Cu-Loaded Cellulose Hydrogel

**Figure 4:**
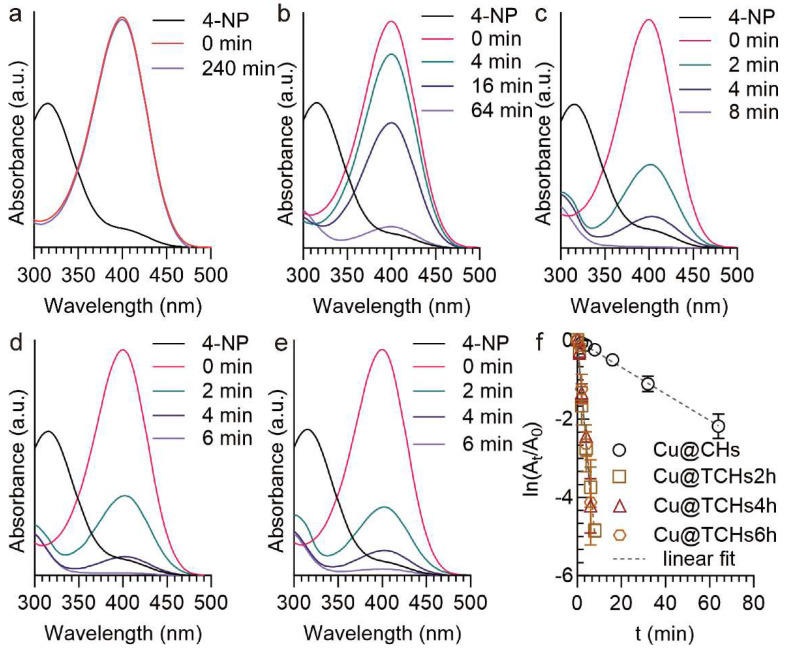
Time-dependent UV–vis absorption spectra of 4-nitrophenol reduction by NaBH_4_ in the (**a**) absence and (**b**–**e**) presence of Cu NP-loaded cellulose hydrogels: (**b**) Cu@CHs, (**c**) Cu@TCHs2h, (**d**) Cu@TCHs4h, and (**e**) Cu@TCHs6h. (**f**) Plots of ln (A_t_/A_0_) against reaction time for catalytic 4-NP Cu-loaded cellulose hydrogels.

The catalytic performance of Cu-loaded cellulose hydrogels was evaluated for the reduction of 4-nitrophenol (4-NP) to 4-aminophenol (4-AP) using NaBH_4_. With the addition of NaBH_4_, the 4-NP is deprotonated to form 4-NP ions, and the absorbance peak shifts from 317 nm to 400 nm [35]. However, the 4-NP absorbance remained unchanged even after 240 min (Figure 4a). When Cu NP-loaded cellulose hydrogels were added, the 4-NP absorbance decreased gradually, and the almost complete conversion of 4-NP to 4-AP was achieved; Cu@CHs was added, and the required times to thoroughly convert 4-NP to 4-AP were found to be over 64 min (Figure 4c). Cu NP-loaded oxidized cellulose hydrogels (Cu@TCHs2h, Cu@TCHs4h, and Cu@TCHs6h) were added, the absorbance of 4-NPs rapidly decreased, and the almost complete conversion of 4-NP to 4-AP was achieved within 8 min.

The linear relationship between ln(A_t_/A_0_) and t, as shown in Figure 4f, suggests that the reduction reaction of 4-NP followed pseudo-first-order kinetics. According to the slope of the fitted curves, the apparent rate constants were determined to be Cu@CHs for 0.0339 min^−1^. The apparent rate constant (k) of Cu NP-loaded oxidized cellulose hydrogels (Cu@TCHs2h for 0.6308 min^−1^, Cu@TCHs4h for 0.6713 min^−1^, and Cu@TCHs6h for 0.6677 min^−1^) increased up to about 20 times compared to that of Cu@CHs. This is because more Cu NPs were loaded onto the oxidized cellulose hydrogels, providing more catalytic active sites, thereby accelerating the reduction reaction of 4-NP. We further measured the catalytic activity from the catalytic dose (different pieces of Cu@TCHs4h) under the same reduction reaction. With the increase in the pieces of the Cu@TCHs4h, the required time to thoroughly convert 4-NP to 4-AP is significantly shortened (Figure S5a–c). The linear relationship between ln(A_t_/A_0_) and t is as shown in Figure S5d. According to the slope of the fitted curves, the apparent rate constants (k) were 0.1729, 0.2864, and 0.6713 min^−1^, for one piece, three pieces, and five pieces of Cu@TCHs4h, respectively. These results indicate that Cu NP-loaded oxidized cellulose hydrogels show excellent catalytic performance for 4-NP reduction.

**Figure 5:**
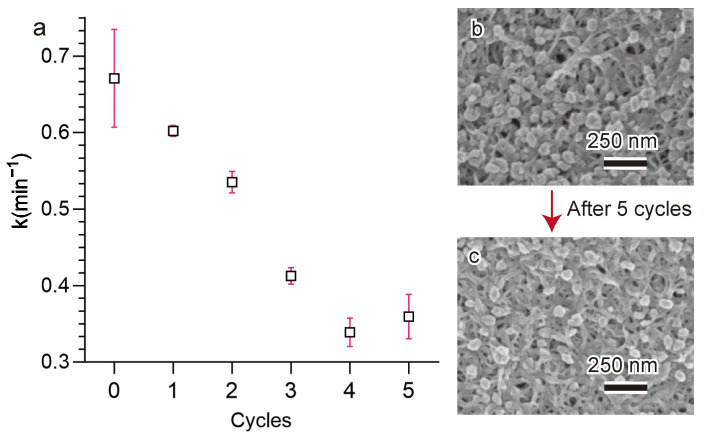
(**a**) Kinetic rate constant for the reduction of 4-nitrophenol by NaBH_4_ catalyzed by a Cu@TCHs4h reusability evaluation of 5 cycles; (**b**) SEM image of Cu@TCHs4h before 5 cycles; and (**c**) SEM image of Cu@TCHs4h after 5 cycles.

The recyclability of catalysts in successive reactions is crucial for practical applications. To evaluate the reusability of Cu@TCHs4h, the same catalyst was reused five times in the 4-NP reduction reaction. After each cycle, the Cu@TCHs4h was washed with distilled water and subsequently reused for the next cycle. In the fifth cycle, the Cu@TCHs4h still demonstrated excellent catalytic performance, as 4-NP could be completely reduced to 4-AP within 14 min (Figure S6), and the reaction rate (k) of Cu@TCHs4h decreased from 0.67 min^−1^ to 0.36 min^−1^ (Figure 5a). This decrease in the k value may have primarily resulted from the loss of Cu NPs from the cellulose hydrogels during recycling. The SEM image of Cu@TCHs4 before and after five catalytic cycles is shown in Figure 5b,c, respectively. The number of Cu NPs decreased significantly after five cycles. We measured the TGA curve of Cu@TCHs4h after five catalytic cycles (Figure S7). The amount of Cu NPs in the Cu@TCHs4h after five cycles (dried cellulose gels) is 15.9%.

**Figure 6:**
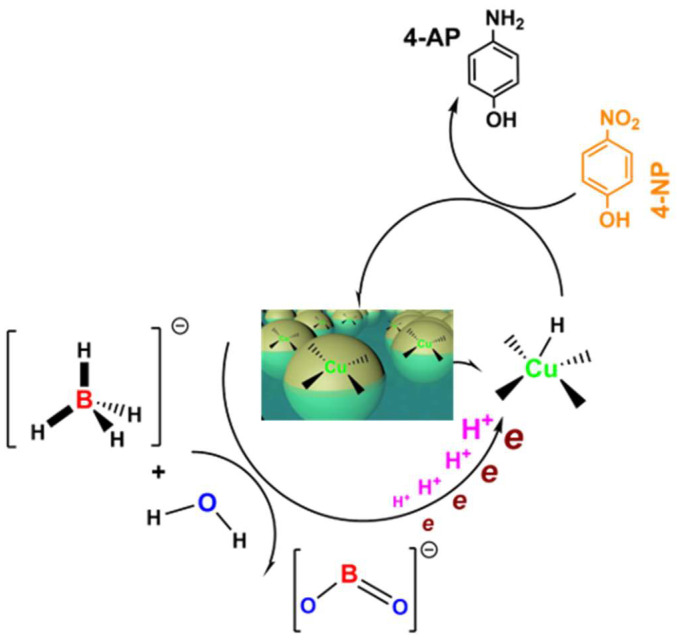
Proposed mechanism for the catalytic reduction of 4-NP in the presence of the Cu NP-loaded cellulose hydrogels and NaBH_4_.

The proposed mechanism for the catalytic reduction of 4-NP in the presence of the Cu NP-loaded cellulose hydrogels and NaBH4 is shown in Figure 6. 4-NP and BH_4_^−^ are adsorbed on the surface of cellulose hydrogels loaded with Cu NPs. BH_4_^−^ reacts with water to generate H^+^ and BO_2_^−^ species, while electrons are transferred from the BH_4_^−^ donor to the Cu active sites. The generated H^+^ is further activated by the negatively charged Cu sites, forming a large number of Cu-H intermediates. Due to the strong reducing ability of the active hydrogen species, they attack the -NO_2_ groups and reduce it to -NH_2_ [36].

**Figure 7:**
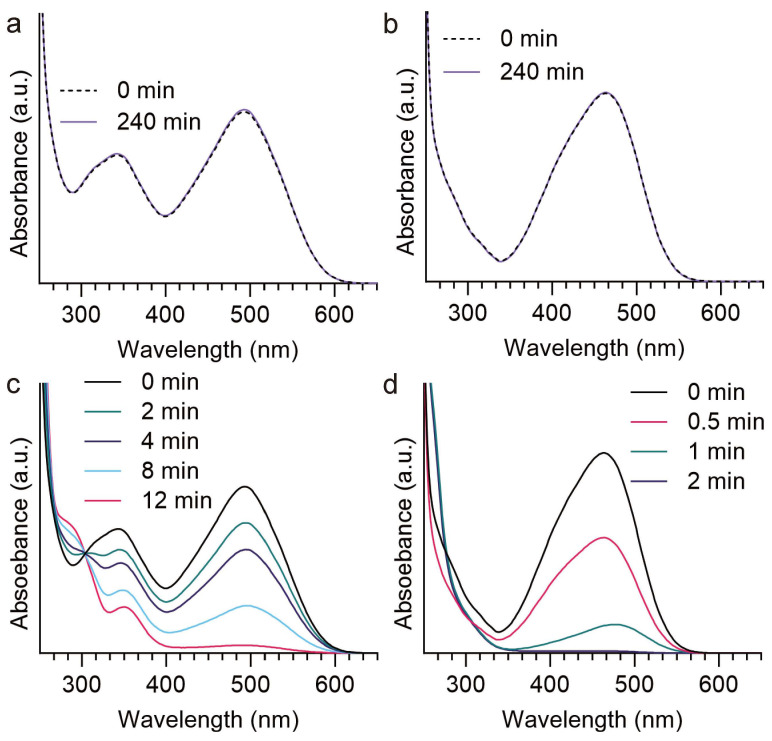
Time-dependent UV–vis absorption spectra of CR and MO by NaBH_4_ in the (**a**,**b**) absence and (**c**,**d**) presence of Cu-loaded cellulose hydrogels.

To investigate the universality of Cu NP-loaded cellulose hydrogels as catalysts for other dyes, MO and CR were selected as test targets to evaluate the catalytic activity of the Cu@TCHs4h catalyst. In the absence of Cu@TCHs4h, the MO and CR absorbance remained unchanged after 240 min (Figure 7a,b). With the addition of Cu@TCHs4h, the absorbance peak at 494 nm (MO) and 464 nm (CR) decreased with reaction time, and these peaks completely disappeared within 12 min and 2 min for MO and CR, respectively (Figure 7c,d). These findings indicate that Cu NP-loaded cellulose hydrogels exhibit excellent catalytic performance and hold broad application prospects in the field of dye degradation.

## 3. Conclusions

In this study, a 60 wt% LiBr aqueous solution was used as a green solvent to successfully prepare cellulose hydrogels through a dissolution–regeneration process. The hydrogels exhibit three-dimensional nanofibril network structure, providing an ideal substrate for the efficient loading of copper NPs. To optimize the catalytic performance, we systematically investigated the effects of TEMPO oxidation reaction times on the carboxyl content on the surface of cellulose hydrogels and the loading behavior of Cu NPs. The results showed that with the extension of TEMPO oxidation time, the carboxyl content on the hydrogels significantly increased, thereby greatly enhancing the anchoring efficiency of Cu NPs. By regulating the oxidation degree, the controlled loading of Cu NPs (10–120 nm) on the surface of the hydrogels was achieved, and the loading amount were significantly better than those of the unoxidized cellulose. Catalytic performance tests demonstrated that the Cu NP-loaded oxidized hydrogels exhibited outstanding activity in the reduction of 4-nitrophenol (4-NP), with an apparent rate constant (k) as high as 0.6 min^−1^, which was 20 times that of the Cu NP-loaded unoxidized hydrogels. Additionally, the catalyst could be directly retrieved using tweezers and remained structurally intact after five cycles of reuse, the reaction rate decreases from 0.67 min^−1^ to 0.36 min^−1^, but it still completed the conversion of 4-NP to 4-AP. Moreover, Cu NP-loaded oxidized hydrogels are also effective in degrading organic dyes such as methyl orange and Congo red, highlighting its broad-spectrum catalytic applicability. This work presents a green, efficient, reusable, and cost-effective catalyst that can potentially replace noble metals for certain catalytic applications. Future studies are required to expand the catalytic application to other reduction reactions and continuous flow systems, and they need to investigate the stability performance of catalysts under various actual conditions such as different pH and ionic strengths. Furthermore, the TEMPO-mediated oxidation reaction may enhance the sensitivity of the hydrogel to environmental humidity [37]. Future research could systematically investigate the influence of relative humidity on the stability of Cu nanoparticles and the catalytic performance of this hybrid hydrogel.

## 4. Materials and Methods

### 4.1. Materials

Regenerated cellulose (Asahi Kasei Co., Ltd., Tokyo, Japan) was used as the starting material. 4-acetamido-2, 2, 6, 6-tetramethylpiperidine-1-oxyl (TEMPO) were purchased from Tokyo Chemical industry Co., Ltd., Tokyo, Japan. Lithium bromide (LiBr), sodium chlorite (NaClO_2_), sodium hypochlorite (NaClO), copper (II) (CuSO_4_·5H_2_O), sodium borohydride (NaBH_4_), 4-nitrophenol (4-NP), methylene orange (MO), and Congo red (CR) were purchased from Woko pure chemical Co., Ltd., Osaka, Japan. Deionized water was used for all experiments.

### 4.2. Preparation of Tempo-Oxidized Cellulose Hydrogels

A total of 1 g of cellulose was added to a 60 wt% LiBr solution. After heating for 20 min at 120 °C, a transparent solution was obtained. The solution was cast onto a glass plate to form a 1 mm-thick layer and was cooled in an air environment. Subsequently, the film was placed in an environment of 70 °C, where cellulose hydrogels (CHs) gradually formed. The cellulose hydrogels were thoroughly washed with deionized water. Forty pieces of 2 cm × 2 cm hydrogel (weighing approximately 0.2 g each) were placed in an Erlenmeyer flask equipped with a magnetic stirrer bar. A total of 200 mL of 0.1 M acetate buffer containing NaClO_2_ (80%, 1.7 g) and TEMPO (0.192 g) was added to the flask. A 1% diluted NaClO solution (0.62 mL) was prepared by diluting with water and subsequently added to the suspension in one step. The flask was sealed with a universal stopper, and the mixture was stirred at 40 °C for 2 h, 4 h, and 6 h. The resulting Tempo-oxidized cellulose hydrogels were denoted as TCHs2h, TCHs4h, and TCHs6h, respectively. The carboxylate content of each oxidized cellulose hydrogel was determined using the conductometric titration method [38]. Additionally, the cellulose hydrogel was stained with 0.01% ruthenium red for 2 h at room temperature to determine the localization of carboxyl groups [38].

### 4.3. Preparation of the Cu NP-Loaded Cellulose Hydrogels

Five pieces of cellulose hydrogel (weighing approximately 25 mg each) were immersed in 15 mL of a 200 mM CuSO_2_·5H_2_O solution and kept overnight. The cellulose hydrogel with adsorbed Cu^2+^ ions was then washed with deionized water and subsequently placed in a freshly prepared NaBH_4_ aqueous solution (15 mL, 0.01 g/mL) under stirring at room temperature for 3 h to reduce the adsorbed Cu^2+^ ions within the cellulose hydrogel. The color of the hydrogel changed from royal blue to black, indicating the formation of Cu NPs. The oxidized cellulose hydrogels loaded with Cu NPs (TCHs2h, TCHs4h, and TCHs6h) were correspondingly denoted as Cu@TCHs2h, Cu@TCHs4h, and Cu@TCHs6h, respectively. The unoxidized cellulose hydrogel loaded with Cu NPs was denoted as Cu@CHs.

### 4.4. Catalytic Performance of Cu NP-Loaded Cellulose Hydrogels

Catalytic degradation of 4-NP by NaBH_4_ was performed using Cu NP-loaded cellulose hydrogel as the catalyst. First, an aqueous solution of NaBH_4_ (20 mL, 0.06 M) was added to a 200 mL conical flask containing an aqueous solution of 4-NP (100 mL, 0.1 mM) under stirring. Subsequently, five pieces of Cu NP-loaded cellulose hydrogel films were added to the above solution at room temperature, and the absorption peak at 400 nm was recorded using a UV–vis spectrophotometer. In the catalytic reduction reactions, since the amount of NaBH_4_ was in excess relative to 4-NP, it is reasonable to assume that the reaction rate was independent of the NaBH_4_ concentration. Thus, this reaction can be considered a pseudo-first-order reaction with respect to 4-NP [39]. This kinetic model can be expressed by the following equation:

$$\ln\frac{A_{t}}{A_{0}}=-\mathrm{kt}$$

where A_0_ is the absorbance of the peak at 400 nm at initial stages. A_t_ is the absorbance of peal at reaction time t, and k is the pseudo-first-order rate constant.

Once the reaction was completed, the Cu-loaded cellulose hydrogels were removed from the solution and thoroughly rinsed with deionized water. The hydrogels were then reused to initiate the next cycle of the reaction. In accordance with similar studies in this field [17,36], we performed five cycles of reusability testing. MO and CR were also employed to investigate the catalytic performance of Cu-loaded cellulose hydrogels, with reaction conditions similar to those for the reduction of 4-NP. The initial concentrations of MO and NaBH_4_ were 0.5 mM and 0.06 M, respectively. The initial concentrations of CR and NaBH_4_ were 0.05 mM and 0.06 M, respectively. The catalytic processes were monitored at 494 nm (CR) and 464 nm (MO) using a UV–vis spectrophotometer [13]

### 4.5. Characterization

The Fourier transform infrared spectroscopy (FTIR) measurements were performed using the ATR method (PerkinElmer, Inc., Waltham, MA, USA). The FTIR spectra were recorded in absorbance mode within the range of 400–4000 cm^−1^ at a resolution of 4 cm^−1^. The carboxylate content of the hydrogel was determined using the electric conductivity titration method with an automatic potentiometric titrator (AT-710S, Shimadzu, Co., Kyoto, Japan). Cross-sectional light-microscope images of the ruthenium red-stained hydrogel were obtained using an IX71 light microscope (Olympus, Co., Tokyo, Japan). The morphologies of the cellulose hydrogel and Cu NP-loaded cellulose hydrogel were further characterized by field emission-scanning electron microscopy (FE-SEM, Hitachi S-4800, Hitachi, Ltd., Tokyo, Japan). All samples for SEM analysis were prepared via solvent exchange (water → ethanol → t-butanol) followed by freeze drying. To enhance conductivity and image contrast, the samples were sputter-coated with a thin layer of osmium (Os) prior to SEM observation. The SEM images were acquired at an acceleration voltage of 1.5 kV. Wide-angle powder X-ray diffraction (XRD) patterns were recorded using a R-AXIS RAPID II diffractometer (Rigaku, Co., Tokyo, Japan) over a 2θ range of 10–70° with Cu Kα radiation (λ = 0.1542 nm) generated at 50 kV and 100 mA. Thermal gravimetric analysis (TGA) was conducted using a TGA-51 instrument (Shimadzu, Co., Kyoto, Japan), where the sample was heated from 35 °C to 800 °C at a heating rate of 10 °C/min under a nitrogen flow of 50 mL/min. The degradation reaction was analyzed using a UV-2450 UV–vis absorption spectrophotometer (Shimadzu, Co., Kyoto, Japan).

## Data Availability

The data supporting the findings of this manuscript are available from the corresponding authors upon reasonable request.

## Supplementary Materials

The following supporting information can be downloaded at: https://www.mdpi.com/article/10.3390/gels11070512/s1, Figure S1: FTIR spectra of raw cellulose and regenerated cellulose gels; Figure S2: The cross-section of unoxidized cellulose hydrogel and oxidized cellulose hydrogels; Figure S3: EDX mapping image of Cu@TCHs4h; Figure S4: TGA curve of the Cu NPs loaded cellulose hydrogels and unloaded cellulose hydrogels, a CHs and Cu@CHs, b TCHs2h and Cu@TCHs2h, c TCHs4h and Cu@TCHs4h, and d TCHs6h and Cu@TCHs6h; Figure S5: Time-dependent UV–vis absorption spectra of 4-nitrophenol reduction by NaBH4 in different pieces of Cu@TCHs4h: (a) 1 piece, (b) 3 pieces, (c) 5 pieces, and (d) Plots of ln (At/A0) against reaction time for the catalytic of 4-NP with different pieces of Cu@TCHs4h; Figure S6: Time-dependent UV–vis absorption spectra for the reduction of 4-nitrophenol by NaBH4 catalyzed by Cu@TCHs4h reusability evaluation 5th cycles; Figure S7: TGA curve of Cu@TCHs4h before and after 5 catalytic cycles.
