# Supplementary material for: TEMPO-Oxidized Cellulose Hydrogels Loaded with Copper Nanoparticles as Highly Efficient and Reusable Catalysts for Organic Pollutant Reduction
Source: Gels. 2025 Jul 1;11(7):512. doi: 10.3390/gels11070512 (PMC12294658; doi:10.3390/gels11070512)
Supplement: Supplementary file 1 [file gels-11-00512-s001.zip › gels-3704337-supplementary.pdf]

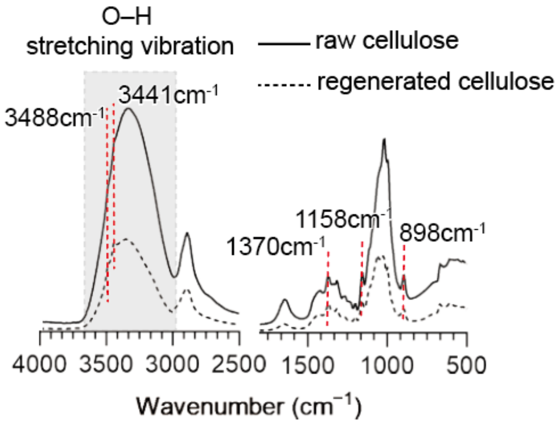

Figure S1. FTIR spectra of raw cellulose and regenerated cellulose gels

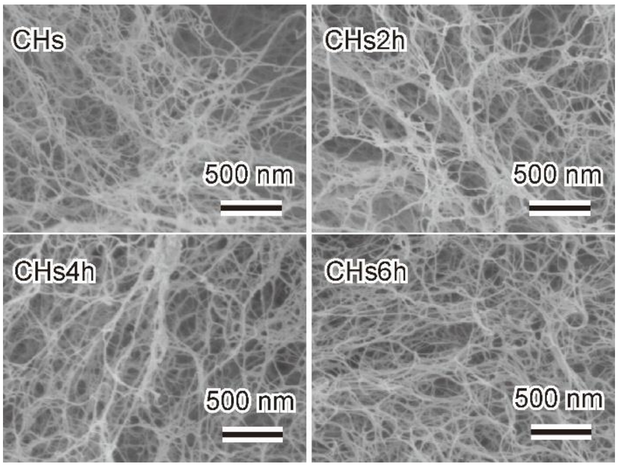

Figure S2. The cross-section of unoxidized cellulose hydrogel and oxidized cellulose hydrogels.

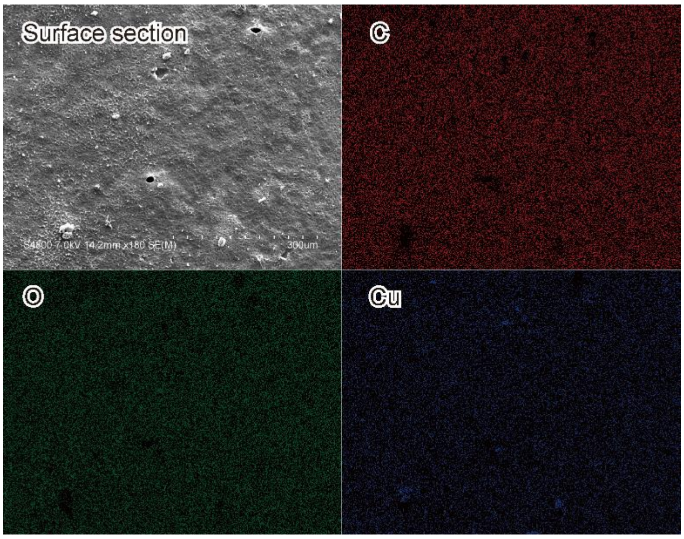

Figure S3. EDX mapping image of Cu@TCHs4h.

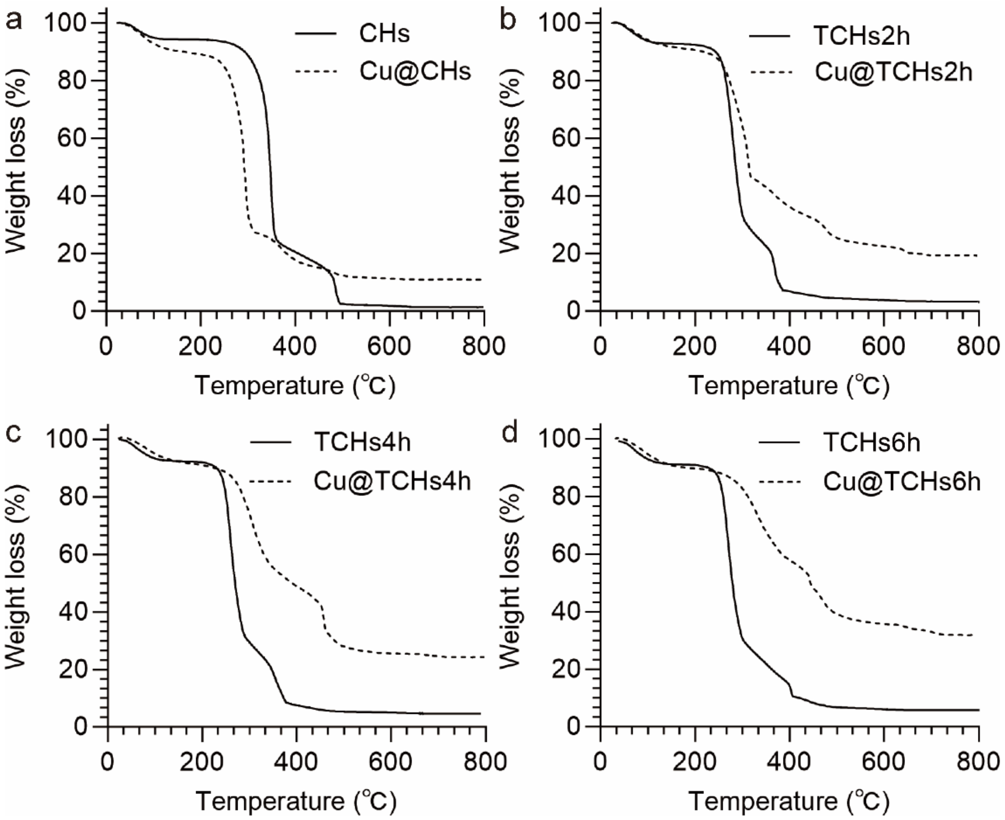

Figure S4. TGA curve of the Cu NPs loaded cellulose hydrogels and unloaded cellulose hydrogels, a CHs and Cu@CHs, b TCHs2h and Cu@TCHs2h, c TCHs4h and Cu@TCHs4h, and d TCHs6h and Cu@TCHs6h.

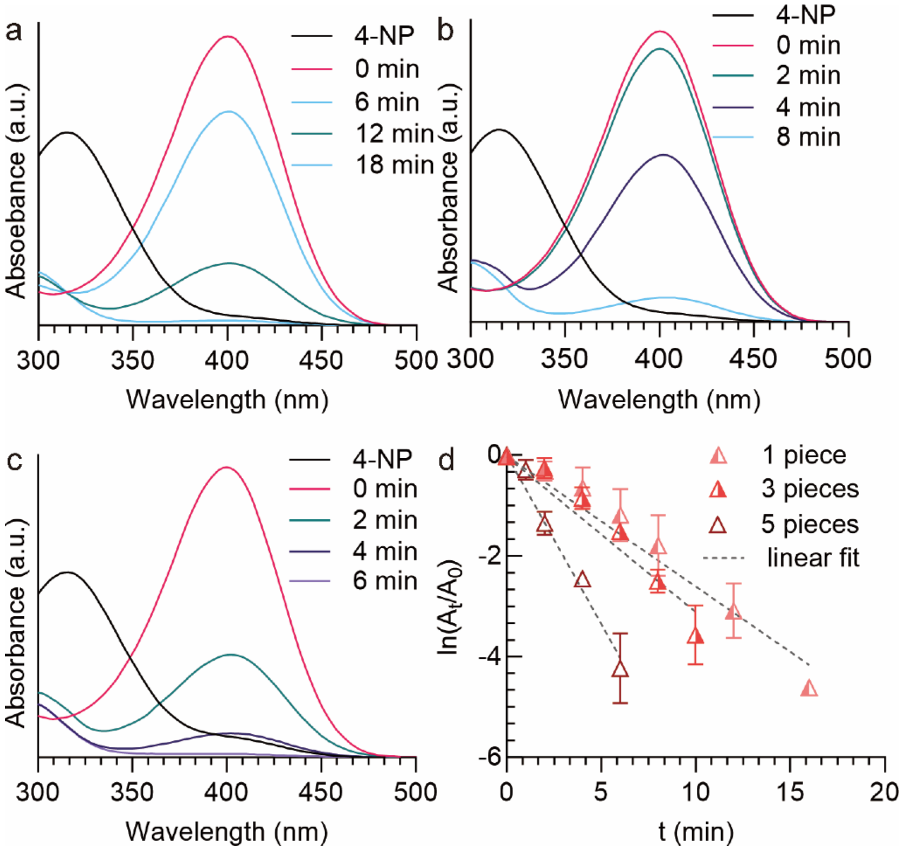

Figure S5. Time-dependent UV–vis absorption spectra of 4-nitrophenol reduction by $NaBH_4$ in different pieces of Cu@TCHs4h: (a) 1 piece, (b) 3 pieces, (c) 5 pieces, and (d) Plots of ln (At/A0) against reaction time for the catalytic of 4-NP with different pieces of Cu@TCHs4h.

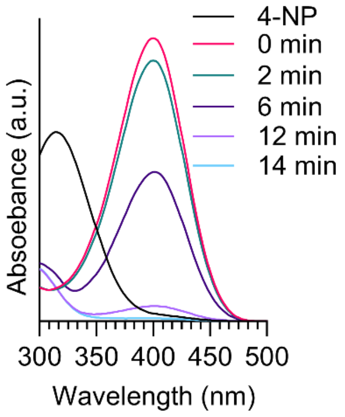

Figure S6. Time-dependent UV–vis absorption spectra for the reduction of 4-nitrophenol by $NaBH_4$ catalyzed by Cu@TCHs4h reusability evaluation 5th cycles.

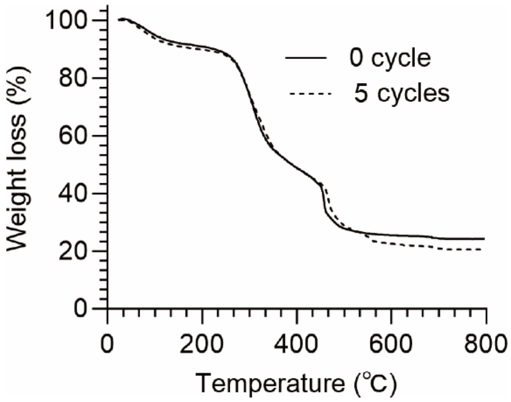

Figure S7. TGA curve of Cu@TCHs4h before and after 5 catalytic cycles.
